# Sustained Release of VEGF to Promote Angiogenesis and Osteointegration of Three-Dimensional Printed Biomimetic Titanium Alloy Implants

**DOI:** 10.3389/fbioe.2021.757767

**Published:** 2021-11-15

**Authors:** Youbin Li, Yuzhe Liu, Haotian Bai, Ronghang Li, Jing Shang, Zhengqing Zhu, Liwei Zhu, Chenyi Zhu, Zhenjia Che, Jincheng Wang, He Liu, Lanfeng Huang

**Affiliations:** ^1^ Orthopaedic Medical Center, The Second Hospital of Jilin University, Changchun, China; ^2^ Orthopaedic Research Institute of Jilin Province, Changchun, China

**Keywords:** 3D-printed porous titanium alloy scaffold, VEGF, angiogenesis, bioactive interface, osseointegration

## Abstract

Tumor resection and treatment of trauma-related regional large bone defects have major challenges in the field of orthopedics. Scaffolds that treat bone defects are the focus of bone tissue engineering. 3D printing porous titanium alloy scaffolds, prepared via electron beam melting technology, possess customized structure and strength. The addition of a growth factor coating to the scaffold introduces a specific form of biological activation. Vascular endothelial growth factor (VEGF) is key to angiogenesis and osteogenesis *in vivo*. We designed a porous titanium alloy scaffold/thermosensitive collagen hydrogel system, equipped with VEGF, to promote local osseointegration and angiogenesis. We also verified the VEGF release via thermosensitive collagen and proliferation and induction of the human umbilical vein endothelial cells (HUVECs) via the composite system *in vitro*. *In vivo*, using microscopic computed tomography (Micro-CT), histology, and immunohistochemistry analysis, we confirmed that the composite scaffold aids in angiogenesis-mediated bone regeneration, and promotes significantly more bone integration. We also discovered that the composite scaffold has excellent biocompatibility, provides bioactive VEGF for angiogenesis and osteointegration, and provides an important theoretical basis for the restoration of local blood supply and strengthening of bone integration.

## Introduction

Bone has a strong regeneration capacity. In fact, small bone defects are often healed by self-repair. Severe trauma, bone tumor resection, and bone infection, however, lead to a large area of bone defect, which exceeds the ability of self-healing. In these cases, we rely on autologous bone repair after artificial intervention (Loi et al., 2016). Moreover, owing to complications like limited amount of autogenous bone, damage of bone graft site, and bone necrosis after transplantation, scientists are continuously searching for effective bone substitutes (Kinaci et al., 2014; Fillingham and Jacobs 2016). In recent decades, the tissue engineering platform used materials science, bioengineering, and stem cell technology to produce bone substitutes. With rising demand in bone repair, bone tissue engineering is an ideal method of promoting bone regeneration and it is gaining ground in clinical practice as well (Bose et al., 2012; Lindsey and Mohan 2016; Thoma et al., 2017; Li et al., 2021). Blood vessels play an essential role in promoting regeneration and repair of segmental bone defects, and lack of blood vessels can result in severe necrosis of the fracture site (Schemitsch 2017; Stegen and Carmeliet 2018). Insufficient blood supply after bone injury is considered to be the main cause of poor fracture healing, affecting rehabilitation in about 10% of fracture patients (Gomez-Barrena et al., 2015). Being an important growth factor in angiogenesis, vascular endothelial growth factor (VEGF) is widely used to induce vascular remodeling (Choi et al., 2013). VEGF increases vascular permeability and promotes angiogenesis. In addition, it is proved to have a strong chemotaxis effect on the migration of endothelial cells. Relevant studies revealed that VEGF is essential for both bone formation and regeneration (Li et al., 2020; Liu et al., 2020; Kauffmann et al., 2021).

Titanium alloys (Ti6Al4V) have high biocompatibility and mechanical strength, making them attractive bone substitutes in both dental and orthopaedic implants. In addition, the corrosion resistance, chemical inertia, and low Young’s modulus of these peptide alloys are additional reasons for their wide application (Sobolev et al., 2019). However, the biological inertia of titanium alloys may inhibit its direct binding to bone tissue after implantation (Williams 2008). Nevertheless, titanium alloy, printed by 3D printing technology, can realize private customization and provide ordered porous structure, which can avoid uneven stress caused by uneven pores and reduce risk of scaffolds damage (Popov et al., 2018). Pores with sufficient diameter can provide adequate space for cell colonization and metabolite transport (Chang et al., 2016). Furthermore, to reduce inertia of the titanium alloy, increase bioactivity, and enhance regeneration of blood vessel and bone within the scaffold, it is necessary to introduce VEGF to the scaffolds.

At present, tissue engineering uses hydrogel as a carrier for cells, drugs, and growth factors. Among them, thermosensitive collagen hydrogel is of particular interest, due to its unique ability to alter from a liquid to a gelatinous state, based on temperature (Patel et al., 2008). Generally, low temperature liquid hydrogel is used to encapsulate cytokines, whereas thermo coagulation colloid is used for sustained drug release. Compared to the systemic drug usage, local drug administration can reduce the amount of drug used, avoid systemic side effects, and improve regional treatment efficacy (Whyte et al., 2008).

In this study, we combined VEGF, thermosensitive collagen hydrogel, and 3D printing titanium alloy scaffolds to construct biologically active surfaces for localized angiogenesis and osseointegration. We hypothesized that the composite scaffold will offer a sustained release of VEGF post implantation, and the released VEGF will recruit rabbit endothelial cells, and enhance bone and vascular regeneration of rabbit distal femoral bone defects.

## Materials and Methods

### Design of 3D Printing Porous Ti6Al4V Scaffold

Porous titanium alloy scaffolds were obtained from EBM Technology (EOS M280, Germany), whose composition is Ti6Al4V. The porosity and pore size of the porous scaffold was 70% and 500 μm, respectively. Porosity= (apparent volume of material in natural state - absolute volume of material)/apparent volume of material in natural state. The diameter of the cylinder was 300 μm. The heights of the cylindrical scaffolds used in cellular and animal experiment were different. The shape of the scaffold for animal experimentation was cylindrical (φ10 × L3 mm), and for cellular experimentation was disc (φ4 × L8 mm) (Li et al., 2018). Relevant experiments confirmed that such parameter design can provide sufficient living space for cells, and the coincidence rate of parameters close to human bone is high (Ran et al., 2018; Arjunan et al., 2020; Zhu et al., 2021). The 3D models of the two differently designed titanium alloy scaffolds were transformed into general language files and entered into the 3D printing machine. Then, the EBM machine synthesized the scaffold using 45–55 μm Ti6Al4V powder (TLS, Germany). The synthesized scaffolds were then washed thrice in ethanol and deionized water for 45 min, and exposure to ultraviolet light for sterilized after autoclaving.

### Preparation of the VEGF-Loaded Composite Scaffold

Based on instructions from the previous study (Mimura et al., 2008), type I collagen, recombinant buffer, and 10X concentrated medium were mixed in an 8:1:1 ratio at 4°C to prepare a temperature-sensitive collagen hydrogel (0.3% type I collagen; Nitta Gelatin, Osaka, Japan) solution. VEGF (Pepro Tech, United States) dissolved in alginate solution was configured as a long-term storage solution, according to the kit instructions. Then, the VEGF solution was introduced to the prepared low-temperature collagen solution to form a complex (VEGF/thermosensitive collagen). The total amount of VEGF contained in each scaffold was 240 ng. The accepted optimal concentration of VEGF for angiogenesis is 25 ng/ml (Bai et al., 2014). At 4°C, the complex was placed in a 48-well cell culture plate, and the pre-cooled titanium alloy scaffold was immersed into it. Finally, the composite scaffold was placed in a 37°C constant temperature incubator for 30 min to transform the temperature-sensitive collagen from a liquid to gelatinous state.

### Swelling and Degradability Evaluation

In order to evaluate the swelling behavior of thermosensitive collagen hydrogel, the thermosensitive collagen hydrogel was freeze-dried and weighed (W_1_) and then added to PBS at 37°C. Take out the hydrogel at the preset time of each day, use the filter paper to gently absorb excess PBS and weigh it (W_2_). The swelling rate is calculated as follows:

$$Swelling\;ratio\left(\%\right)=\left(W_{2}-W_{1}\right)/W_{1}\times 100\%$$

The degradation behavior of thermosensitive hydrogel in scaffold pores was evaluated by *in vitro* degradation experiments. According to the above method, the composite scaffold was immersed in PBS containing type I collagenase (sigma) at 37°C for degradation experiment. First, the hydrogel was weighed (W_1_), then the gel was taken off every other day at a predetermined time point and weighed (W_t_). The degradation rate of hydrogel was evaluated according to the weight of residual hydrogel. The degradation rate formula is as follows:

$$\text{Degradation}\;\text{ratio}\left(\%\right)=\left(W_{1}-W_{t}\right)/W_{1}\times 100\%$$

### Evaluating Thermosensitive Collagen-Containing Composite Scaffold Microstructure and VEGF Release *in vitro*

The microscopic characteristics and structure of the Ti6Al4V scaffold and the composite scaffold containing thermosensitive collagen were observed via scanning electron microscope (SEM; Shimadzux-550, Japan). In order to obtain the release efficiency of VEGF, composite scaffolds were placed in 1 ml phosphate-buffered solutions (PBS; Solar Beijing, China) at 37°C for varying durations of time, namely, 1 h, 3 h, 8 h, 1 day, 3, 7, 10, and 15 days, and the amount of VEGF release was measured. VEGF levels in the leaches were determined using enzyme-linked immunosorbent assay (ELISA; R&D Systems) and absorbance values were determined by BioTek Instruments (United States) at 450 nm wavelength.

### *In vitro* Cell Experiments

#### Cell Viability

To detect cellular activity and proliferation, live/dead cell staining (BestBio, Shanghai, China) and CCK-8 assay (Bioss, Beijing, China) were performed. As described in previous experiments (Jin et al., 2019), the medium required for the experiment was endothelial cell medium (Sciencell Research Laboratories, China). Empty titanium (eTi), hydrogel-bound titanium (cTi), and hydrogel-bound titanium scaffolds loaded with VEGF (cTi/VEGF) were placed into 24-well plates. Next, 2 ml medium suspension containing human umbilical vein endothelial cells (HUVECs; 5×10^3^/well; Cellcook Biotech, Guangzhou, China) was added to each group of scaffolds. To conduct live/dead cell staining, HUVECs were cultured for 1 and 3 days. Next, the scaffolds with HUVECs were dipped into a working live/dead cellular staining solution, obtained according to manufacturer’s instructions. The immersed sample was then incubated at room temperature in darkness for 15 min. Following that, the working solution was removed via two washes with PBS and the stained HUVECs were observed under a fluorescence microscope. CCK-8 assay, on the other hand, was evaluated after 1, 4, and 7 days of HUVECs co-culture with scaffolds. Briefly, the CCK-8 assay solution was added to each well and incubated at 37°C and 5% CO_2_ for 2 h. Absorbance was then measured at 450 nm using a Bio-Rad microplate reader.

#### Tubule Formation Assay

The matrigel matrix (BD, United States), 96-well plate, and gun head were pre-cooled at 4°C for 12 h before the experiment. 50 μL matrigel matrix was then poured into a 96-well plate, and allowed to form into gel at 37°C for 30 min. Extracts of the eTi, cTi, and cTi/VEGF scaffolds were then used to resuspend the HUVECs. HUVECs (2×10^4^ cells/well) were inoculated on the surface of Matrigel. After 24 h of culture, images were taken under an inverted microscope (Olympus Corporation, Japan). The results were quantitatively analyzed using the ImageJ software (NIH, Bethesda, MD).

#### Cell Invasion Assay

HUVECs were starved and cultured for 12 h before cell lysis and centrifugation. The cells were then resuspended in serum-free medium and 100 μl (2×10^4^/well) of the cell suspension was added to the top layer (Transwell; Corning). Three groups of scaffolds were added to the lower chamber, and 400 μl complete medium containing FBS was added to each lower chamber. The combined 24-well plates were incubated in an incubator at 37°C and 5% CO_2_ for 24 h and subsequently fixed in paraformaldehyde for 20 min before three washes with PBS. Next, the remaining cells in the upper chamber were wiped with a cotton swab. The cells in the lower chamber were stained with crystal violet for 10 min, before observation under an inverted microscope.

#### Wound Healing Assay

HUVECs were seeded in six-well cell culture plates (1×10^6^/well; Corning, New York) and incubated for 12 h. Next, a pipette was used to introduce scratches of the same width in the monolayer of HUVECs. The cells were then washed thrice with PBS, before the addition of serum-free medium and scaffolds. The 6-well plate was then incubated at 37°C and 5% CO_2_ for 24 h, and the wound healing assessment was done under a microscope and the ImageJ software was used for quantitative analysis.

#### RT-qPCR

To observe the effect of VEGF release from composite scaffolds on the maturation and survival of HUVECs, the expressions of Metalloproteinase-2 (MMP-2), Bcl-2-Associated X (Bax), and B-cell lymphoma-2 (Bcl-2) were evaluated via RT qPCR. In short, cells were cultured in 6-well plates (1×10^6^/well) for 1 and 4 days, and RNA was isolated using TRIzol reagent (Invitrogen, Carlsbad, CA, United States). The primer sequences were designed by the primer premier software (PREMIER Biosoft, Palo Alto, CA, United States) and are summarized in Table S1. mRNA was amplified and quantitatively analyzed using Q SYBR green Supermix (Bio-Rad, Hercules, CA, United States) and a QuantStudioTM seven Flex real-time PCR system (Applied Biosystems, Carlsbad, CA, United States). Relative mRNA expression was measured using the 2^−ΔΔCt^ method and was normalized to endogenous GAPDH levels.

### Animal Experiments

To determine whether cTi/VEGF promotes angiogenesis and osteointegration, 78 6-month-old New Zealand male white rabbits (weight 3.5 ± 0.3 Kg) were divided into three groups: eTi, cTi, and cTi/VEGF. Rabbits were anesthetized with 3% Pentobarbital, at a dose of 55 mg/kg. According to the size of the *in vivo* implantation, a cylindrical bone defect, with a radius of 0.3 cm and a height of 1.0 cm, was polished on the lateral epicondyle of the femur. After washing with normal saline, the three groups of scaffolds were randomly placed into the defect. An absorbable suture was placed and to combat disinfection, penicillin (1.5 mg/kg) was injected intramuscularly for 3 days after operation. At weeks 6 and 12 after operation, rabbits were anesthetized and 5 ml of air was injected into their hearts. Next, the distal femur was taken and the soft tissue was harvested and fixed in 4% paraformaldehyde for follow-up experiments.

#### Microscopic Computed Tomography (Micro-CT) Analysis

Micro-CT was used to estimate the inward growth of the bone into the scaffold. A sample was placed into the system and examined at 48 kV voltage, 200 μA current, and 18 μm Image pixel size. 3D visual reconstruction (Skyscan 1,076 Scanner, Bruker Micro-CT, NV, Kontich, Belgium) was carried out on the scanned images. Subsequently, image analysis was done using a bone analyzer (CT analyzer 1.17.7.2 software, Kontich, Belgium). The size of region of interest (ROI) used in Micro-CT analysis is the area where the scaffold is located. Bone volume fraction (BV/TV), trabecular number (Tb.N), trabecular thickness (Tb.Th) and trabecular separation (Tb.Sp) were then determined from the acquired images.

#### Histological Evaluation

The samples were immobilized in 4% paraformaldehyde solution for 2 weeks before decalcification for 1 month. The samples were then dehydrated in a series of fractionated ethanol solutions. After vitrification with xylene, the samples were embedded in paraffin wax and cut into 30 μm thickness for VG staining. The inward bone growth was observed via an inverted microscope (DSX 500; Olympus Corporation, Tokyo, Japan), and ImageJ was used to analyze the surface area of the newly formed bone tissue.

#### Push-Out Test of Samples

The shear strength of each bracket was evaluated via the pushout test. The soft tissue surrounding each bone sample was removed and washed to reveal the scaffold position. Remove large soft tissue with scissors. The remaining small amount of soft tissue was removed by rubbing with sandpaper. A hydraulic testing machine (MTS Mini Bionix, Minneapolis, United States) was used to press the position of the scaffold at a speed of 1 mm/min that separated the scaffold from the bone tissue. The maximal push force of the scaffold at the beginning of disengagement from bone tissue was then recorded, analyzed, and evaluated (n = 3).

#### Immunofluorescence Staining

The specimens were fixed in 4% paraformaldehyde for 2 weeks, before decalcification for 1 month. After the removal of the scaffolds, the remaining bone tissues were sectioned for immunohistochemical analysis. In short, sections were made after 3% H_2_O_2_ treatment. Then, they were sealed with serum, incubated with primary antibody and then, secondary antibodies, stained, counterstained, and dehydrated. Finally, an optical microscope was used for observation at a magnification of ×200.

### Statistical Analysis

Data are presented as mean ± standard deviation. Each group of data was obtained from three independent experiments. Differences between multiple experimental groups were statistically analyzed via one-way analysis of variance (ANOVA) and Tukey multiple comparison test. GraphPad prism v. 8.2 was utilized for all statistical analysis. *p* < 0.05 indicated significant difference.

## Results

### Characteristics of the Composite Scaffold

#### Characterization of the Composite Scaffold

**FIGURE 1:**
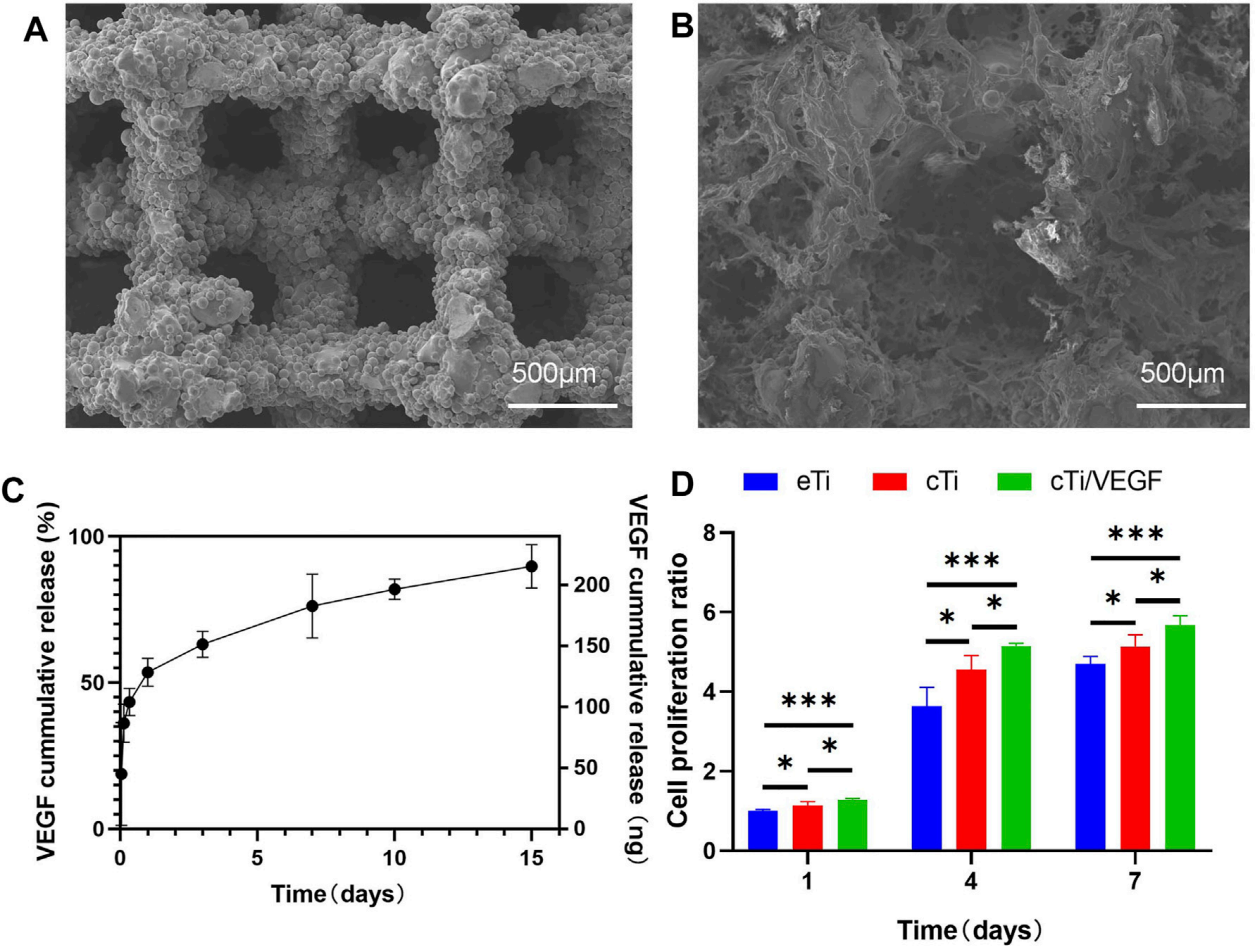
Microscopic characterization of eTi **(A)** and cTi **(B)**, using SEM scanning images **(C)** VEGF release curve *in vitro* within 15 days **(D)** Analysis of the HUVECs proliferation in each group, using the eTi of the first day as a reference (n = 3, *indicates significant differences between groups, *p < 0.05; **p < 0.01; ***p < 0.001).

As shown in Supplementary Figure S1A, the height of the disk-shaped eTi was 3 mm and the diameter was 10 mm. Additionally, the height of the cylindrical eTi was 8 mm and the diameter was 4 mm. The pore arrangement allowed the liquid hydrogel to fill the scaffold more efficiently (Supplementary Figure S1B). The liquid temperature-sensitive collagen from 4°C was left standing at 37°C for 30 min to form a gel (Supplementary Figure S1C). As shown in Figure 1, SEM showed that the pore diameter of the scaffold was 500 μm and the porosity was about 70%. The surface of the scaffold obtained by electron beam melting of Ti6Al4V powder is rough, not flat, and has micron roughness under SEM. Moreover, the surface of the thermosensitive collagen hydrogel exhibited irregular pore arrangement and collagen fiber structure. The diameter of the pores of the hydrogel was about 170 μm and the porosity is about 40%. The thermosensitive collagen hydrogel completely covered the titanium alloy scaffold, and there were multiple continuous micropores in the pores of the scaffold, which provided added space for cellular growth and reproduction.

#### Release Rate of VEGF in Thermosensitive Collagen

As shown in Figure 1C, the release kinetics of cTi/VEGF was recorded over 15 days. On the first day, the VEGF loss rate was 53.5 ± 2.2%. Following a massive release on the first day, the release rate gradually decreased and tapered off in subsequent days, indicating that the thermosensitive collagen hydrogel effectively controlled the release rate of VEGF. On day 15, the VEGF release rate was 89.8 ± 3.4%.

#### Swelling Ability and Degradation Behavior of Hydrogel

As shown Supplementary Figure S2A, thermosensitive collagen hydrogel reached the swelling equilibrium state at 10 h. The swelling ratio is up to 110%. In this experiment, the degradation behavior of hydrogel was observed in PBS containing collagenase I. The degradation rate of the thermosensitive collagen hydrogel in the scaffold pores is shown to be slow degradation in PBS, resulting in a gradual decrease in residual weight (Supplementary Figure S2B). The degradation process of hydrogel can be up to 14 days.

### Cellular Experiments *in vitro*

#### HUVECs Viability

To evaluate whether cTi/VEGF affects proliferation of HUVECs, we performed the CCK-8 assay and analyzed cellular proliferation after inoculation with eTi, cTi, and cTi/VEGF (Figure 1D). The eTi, cTi, and cTi/VEGF cell numbers showed an increasing trend within the first 7 days, but their growth rates were different. On day 1, 4, and 7, the cellular growth rate of cTi/VEGF was higher than the other two groups (*p* < 0.05).

**FIGURE 2:**
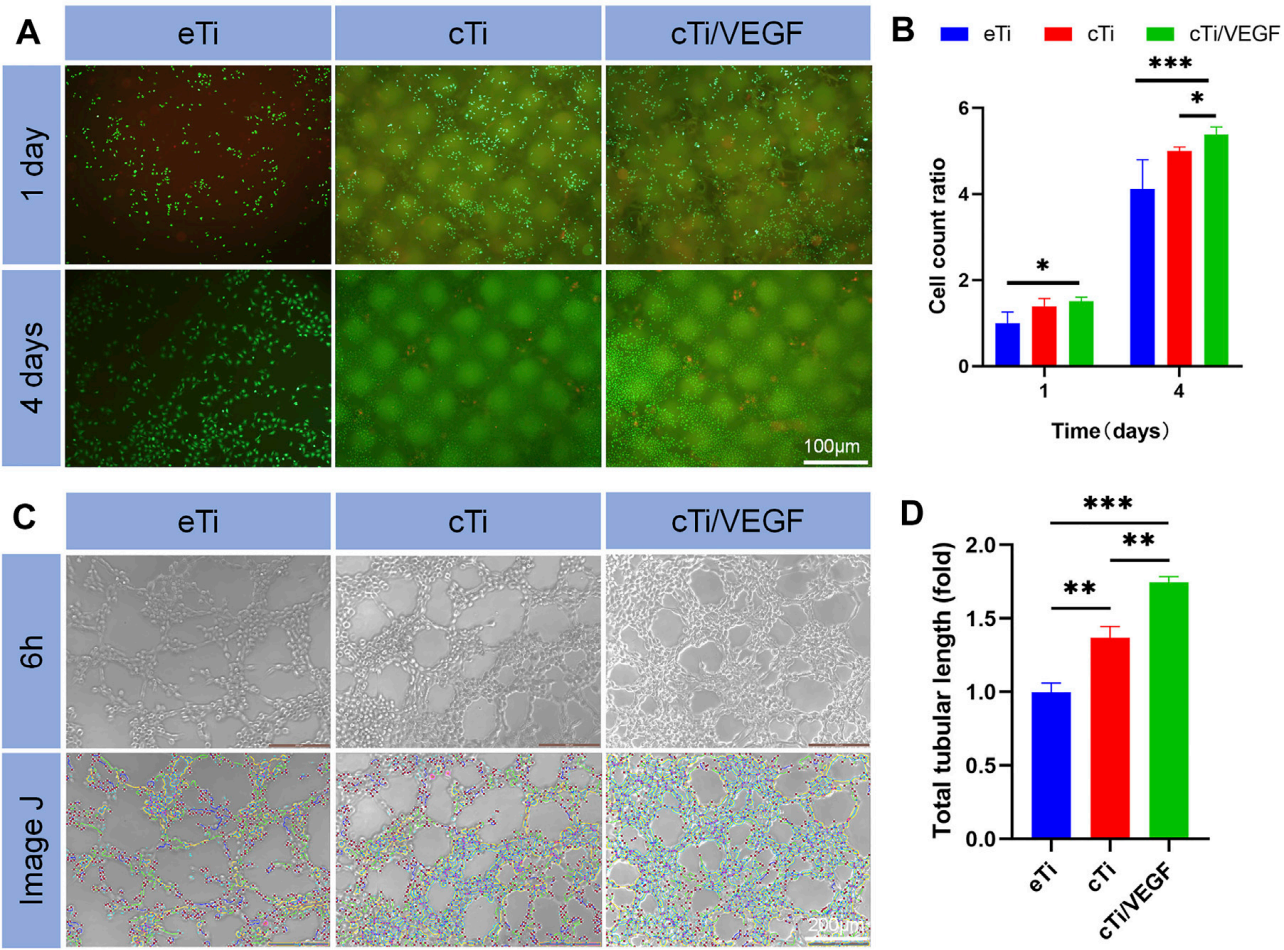
**(A)** Live/dead HUVECs staining was done in a co-culture of HUVECs with eTi, cTi, and cTi/VEGF for 1 and 4 days, respectively (n = 3) **(B)** Total cellular count was evaluated via gross observation, under an inverted microscope **(C)** Tube formation of HUVECs cultured with Extracts of eTi, cTi, or cTi/VEGF for 6 h (n = 3) **(D)** The total tubular lengths of HUVECs in different scaffolds.

Furthermore, the biocompatibility of cTi/VEGF was evaluated via live/dead cellular staining (Figure 2A, B). Compared to the first day, the number of cells in each group increased on the third day. At the same time, we observed that a large number of cells survived in the area covered by collagen, which supported the idea that collagen provides additional attachment points for cellular growth. Moreover, the number and activity of cTi/VEGF cells were much higher than those of the other two groups, indicating that cTi/VEGF has good biocompatibility (*p* < 0.05).

#### Evaluation of Tubule Formation *in vitro*

After 6 h of culture, HUVECs formed lumen-like structures that were analyzed by ImageJ. The results showed that the total length of tubules formed by cTi and cTi/VEGF was longer than that of eTi (Figure 2C). The tubule length of cTi/VEGF was 1.74 ± 0.05-fold larger than eTi, whereas, cTi was 1.37 ± 0.08-fold larger than eTi (Figure 2D). However, the presence of collagen improved the angiogenic activity of the single scaffold. Based on these results, cTi/VEGF can effectively promote tubulogenesis *in vitro* (*p* < 0.05).

#### Evaluation of Cellular Migration Ability

**FIGURE 3:**
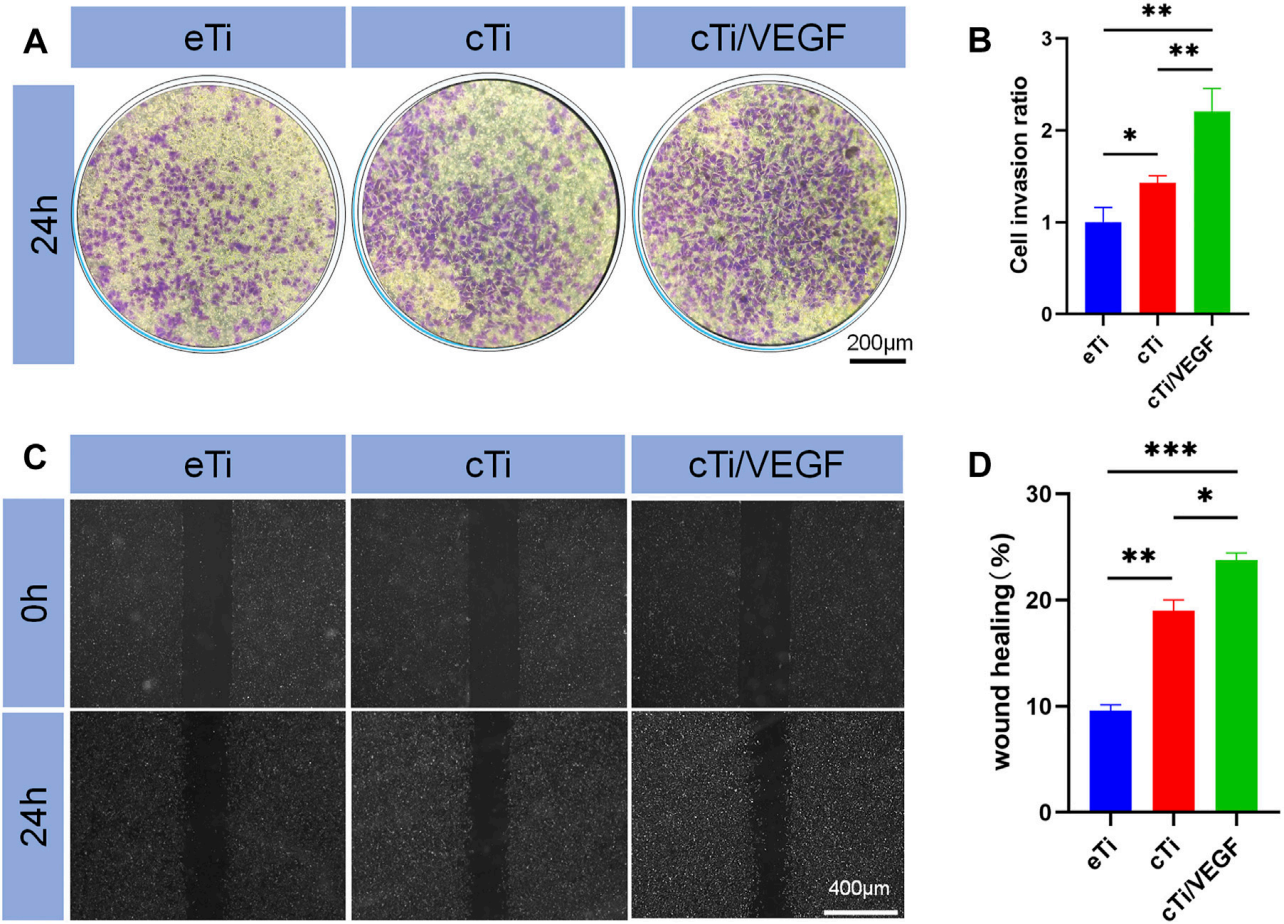
**(A)** Effects of eTi, cTi, and cTi/VEGF on HUVECs invasion **(B)** HUVECs stained with crystal violet were quantitatively analyzed (n = 3) **(C)** The effects of eTi, cTi, and cTi/VEGF on wound healing, as observed under a phase contrast microscope **(D)** Assessment of wound healing rates (n = 3).

The effects of eTi, cTi, and cTi/VEGF on the migration ability of HUVECs were analyzed via invasion and wound healing assays. The ratio of cellular migration and cTi/VEGF was much higher than other groups (Figure 3A).

Assessment of the penetration rate revealed that cTi/VEGF was 1.21 ± 0.27-fold than eTi and 0.78 ± 0.19-fold than cTi (Figure 3B). These results showed that cTi/VEGF possesses good chemotaxis toward cells, and can attract cells to penetrate the membrane. Based on our results from the wound healing assay, cTi/VEGF produced the largest mobility of HUVECs, relative to other scaffolds (Figure 3C). Moreover, the repair rate of eTi was 9.6 ± 0.6%, whereas, that of cTi/VEGF was 23.7 ± 0.7% (Figure 3D). Given that cTi/VEGF produced the largest mobility of HUVECs, we confirmed that cTi/VEGF can improve the migration ability of cells.

#### RT-qPCR

**FIGURE 4:**
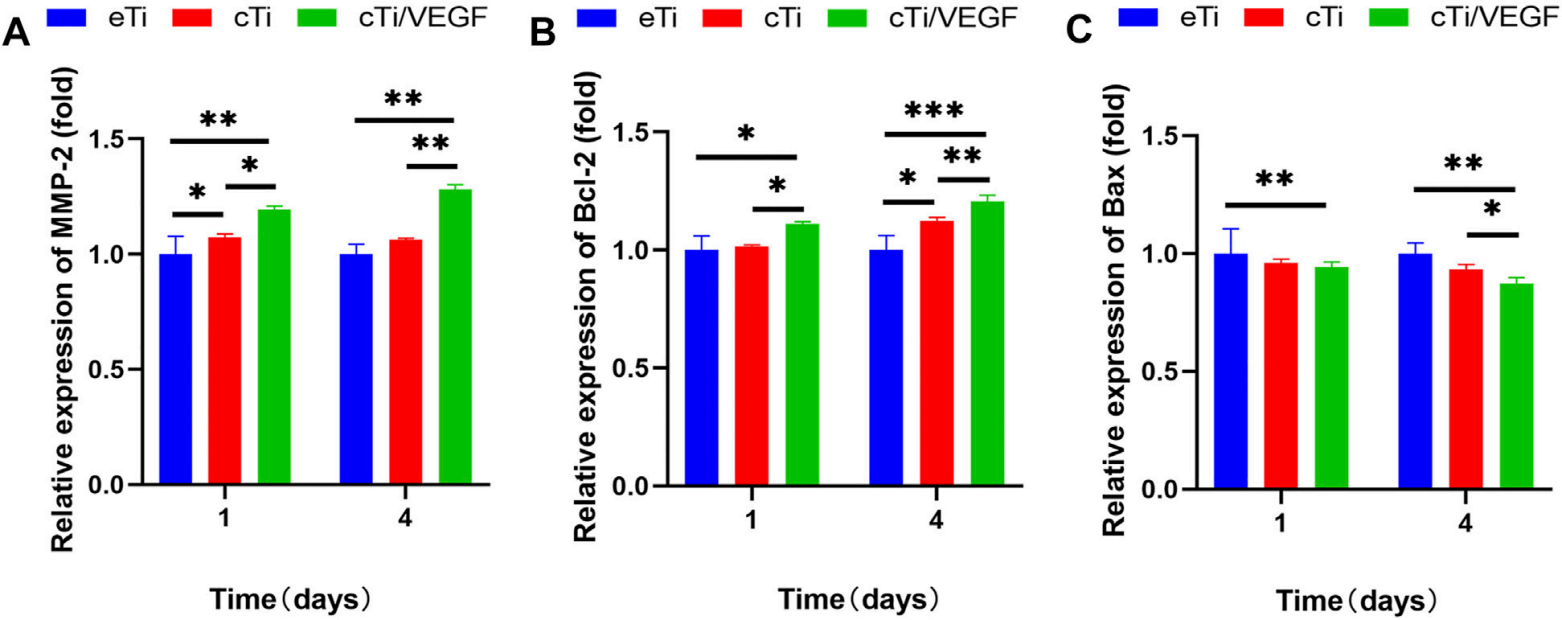
RT-qPCR analysis of **(A)** MMP-2 **(B)** Bcl-2, and **(C)** Bax gene expression in HUVECs cultured with eTi, cTi, and cTi/VEGF for 1 and 4 days, respectively (n = 3).

RT-qPCR was used to evaluate the effects of cTi/VEGF on the maturation and apoptosis of HUVECs. MMP-2 is a marker of angiogenesis *in vitro* (Bai et al., 2014). As shown in Figure 4A, the MMP-2 expression in HUVECs of the cTi/VEGF group was significantly higher than that of HUVECs of the eTi and cTi groups (*p* < 0.05). The MP-2 expressions in cTi/VEGF on day 1 and day 4 were 1.15 ± 0.06 and 1.28 ± 0.02-fold than eTi, respectively. Bax is a pro-apoptotic protease, whereas, Bcl-2 can antagonize cell apoptosis. The day 1 and 4 Bcl-2 expression in cTi/VEGF was increased by 0.11 ± 0.03 and 0.09 ± 0.05 fold, respectively, compared to cTi, and by 0.11 ± 0.09 and 0.21 ± 0.09 fold, respectively, compared to eTi (Figure 4B). Moreover, on day 1 and 4 of culture, Bax of cTi/VEGF was reduced by 0.06 ± 0.06 fold and 0.13 ± 0.08 fold, respectively, compared to eTi (Figure 4C). These results demonstrate that HUVECs possess strong angiogenic activity in cTi/VEGF, and the MMP-2 expression increase gradually. With a prolonged culture time, the expression of anti-apoptotic genes within cTi/VEGF increase as well, while the expression of pro-apoptotic genes decrease.

### *In vivo* Animal Experiments

To evaluate the therapeutic effect of the scaffolds *in vivo*, eTi, cTi, and cTi/VEGF were implanted into the lateral condyles of rabbit femurs (Supplementary Figure S3). All rabbits survived without infection, mental disorders, or other complications. Specimens were removed at 6 and 12 weeks after operation and fixed in 4% paraformaldehyde.

**FIGURE 5:**
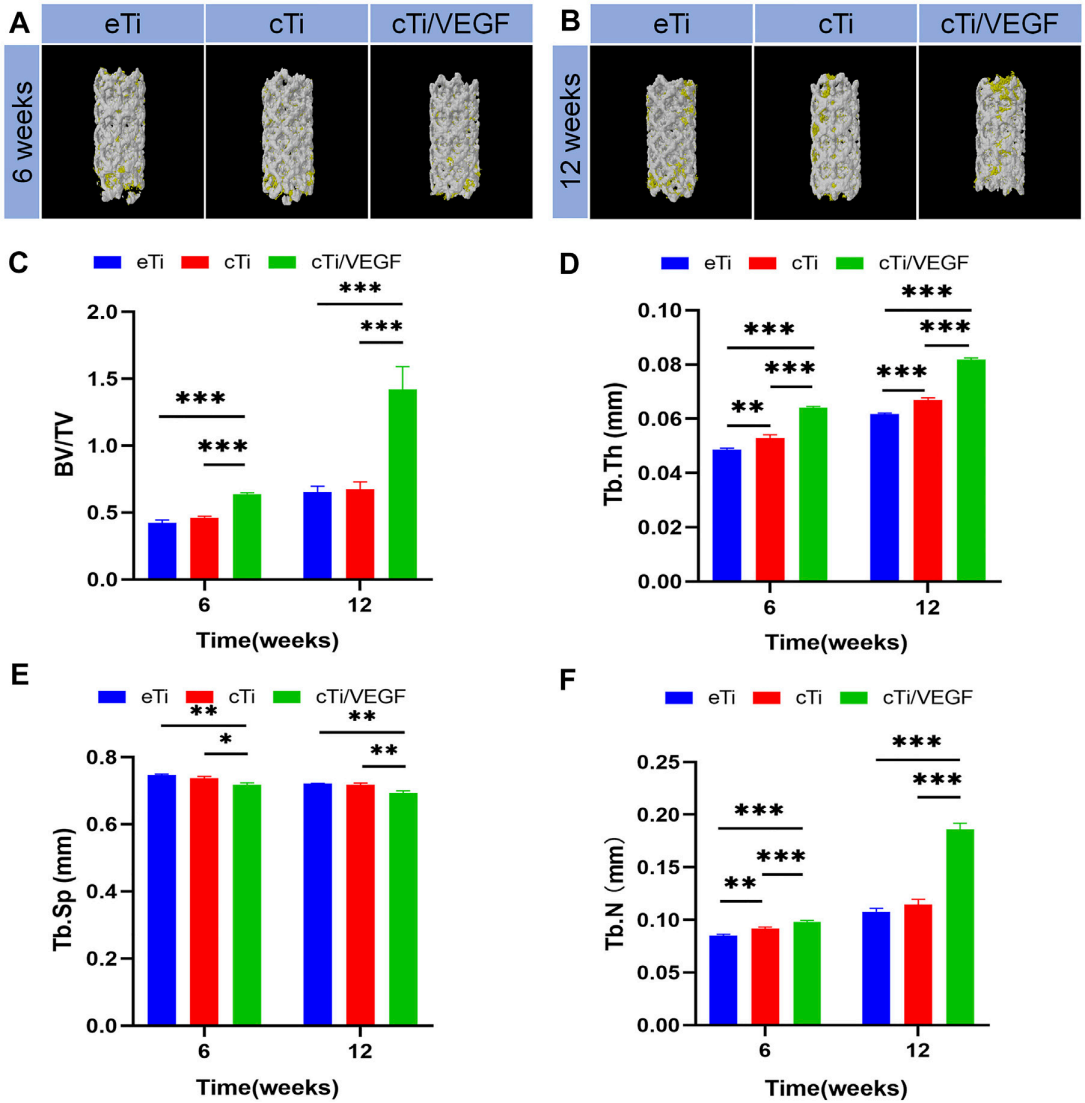
Representative 3D reconstruction images of scaffold implantation in each group at 6 **(A)** and 12 **(B)** weeks after operation (yellow: bone tissue; white: scaffolds). Micro-CT image analysis of BV/TV **(C)**, Tb.Th **(D)**, Tb. Sp **(E)**, and Tb.N **(F)** in each group (n = 3).

Based on our analysis, cTi/VEGF had the best local repair effect, while eTi displayed the worst (Supplementary Figure S4). Micro-CT analysis was performed at 6 and 12 weeks after operation to assess new bone formation around the scaffold in eTi, cTi, and cTi/VEGF implanted rabbits. After 3D reconstruction, Micro-CT showed no significant difference in bone regeneration between eTi and cTi at week 6 after operation (Figure 5A). Conversely, at 6 and 12 weeks after operation, the bone volume of cTi/VEGF was far greater than in eTi and cTi (Figure 5B). Moreover, at 6 and 12 weeks after operation, the bone volume fraction (BV/TV), Tb.Th, and Tb.N were significantly higher in the cTi/VEGF group than in other groups (Figures 5C,D,F). In addition, the Tb. Sp of cTi/VEGF was the lowest at each time point (Figure 5E). These Micro-CT results demonstrated that cTi/VEGF can effectively promote bone regeneration *in vivo*.

**FIGURE 6:**
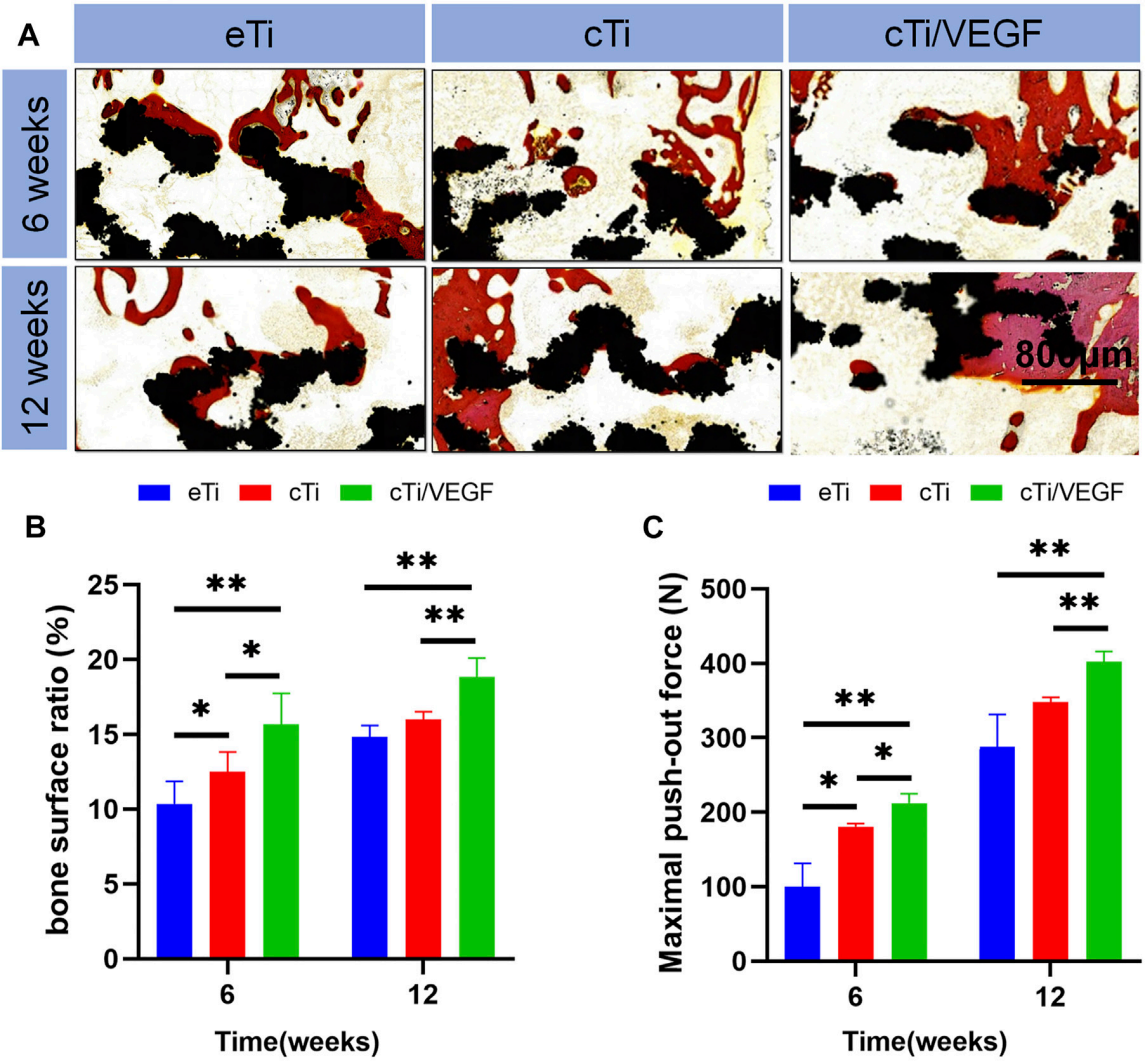
**(A)** The eTi, cTi, and cTi/VEGF scaffolds were separately implanted into rabbit femoral epicondyles and allowed to remain for 6 and 12 weeks. Subsequently, hard sections were prepared and stained with VG (black: Ti6Al4V scaffold; red: new bone) **(B)** the proportion of new bone area to scaffolds pores **(C)** maximum load in the detaching test of eTi, cTi, and cTi/VEGF after implantation into rabbit femoral epicondyles for 6 and 12 weeks (n = 3).

In order to evaluate the bone regeneration inside and around the scaffold, specimens were harvested at 6 and 12 weeks to collect hard tissue sections, and the sections were stained with VG staining (Figure 6A). At 6 weeks after operation, only a small amount of newly formed bone was observed in the scaffold hole in the eTi group. In contrast, a considerable amount of new bones formed in the scaffold of the cTi/VEGF group. At 12 weeks after operation, the amount of bone formation on surfaces and pores in each group increased. The percentage of new bone tissue in the area of the scaffold hole is an important index of bone regeneration measurement. The new bone percentage at 12 weeks after surgery was considerably larger than at 6 weeks post operation, and the proportion of bone in the cTi/VEGF group was the largest (Figure 6B). Moreover, the change in the proportion of bone calculated by ImageJ was consistent with the results of 3D reconstruction.

These results further confirmed that cTi/VEGF promoted bone regeneration and osteointegration at the interface of scaffolds. To gain a better understanding of the degree of integration between the scaffold and bone tissue, we carried out the push-out test (Figure 6C). At 6 and 12 weeks after surgery, the maximum push force of the eTi group was significantly lower than that of the cTi/VEGF group. This suggests that cTi/VEGF accelerated the integration of scaffold and bone and maintained the stability of implantation for a prolonged time.

**FIGURE 7:**
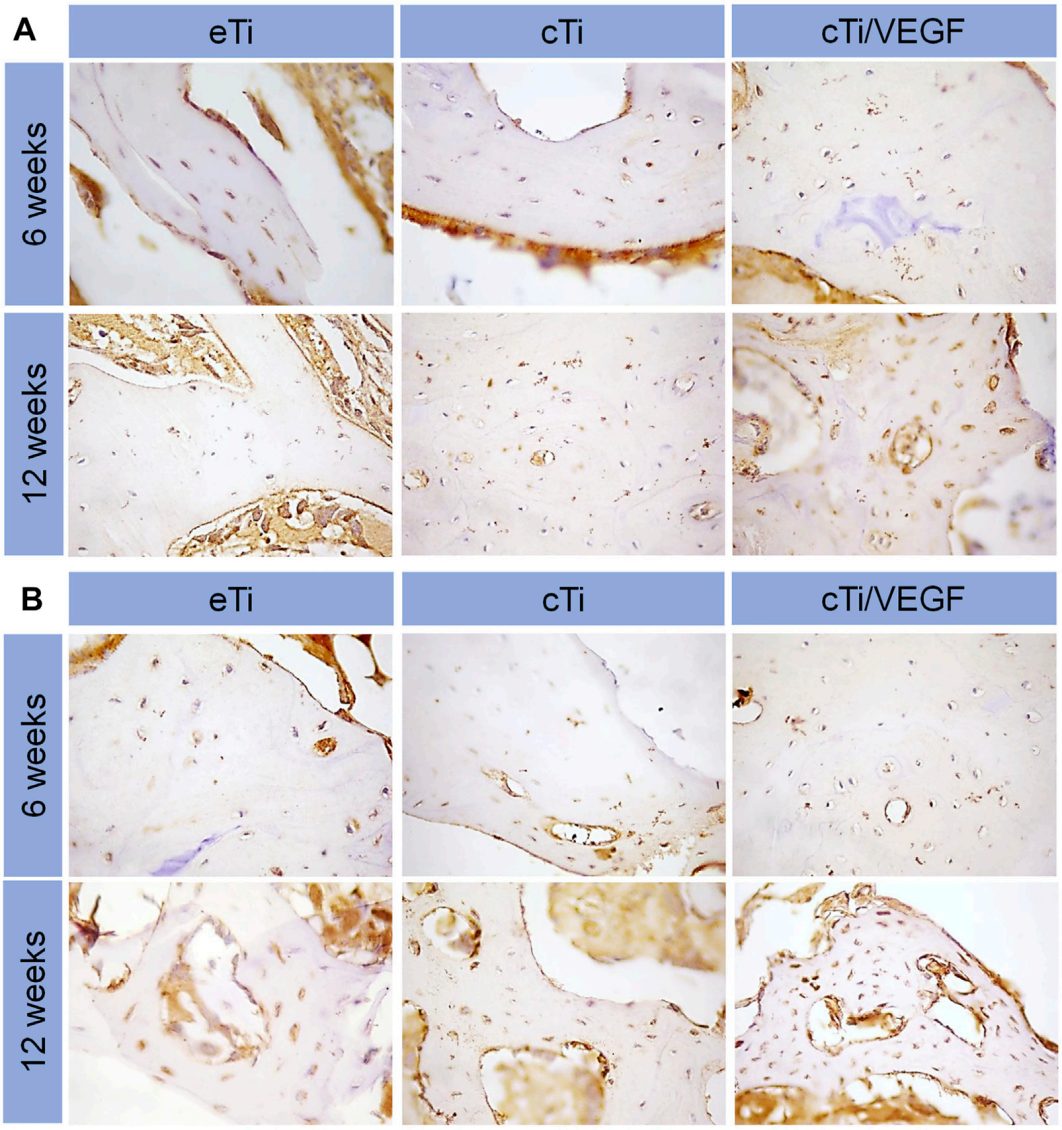
Immunofluorescence staining of COL I **(A)** and CD 31 **(B)** expression in bone surrounding scaffolds of eTi, cTi, and cTi/VEGF after implantation for 6 and 12 weeks (n = 3).

To further examine angiogenesis and osteointegration of the composite scaffolds, we performed immunohistochemical staining on the tissues around the scaffolds. Type I collagen is an important marker of bone regeneration, and it forms bone through mineralization and deposition. In the cTi/VEGF group, we observed significant new bone formation and bone trabecular structure around the scaffold. These results indicate that the cTi/VEGF group has enhanced osteointegration in the 3D-printed porous titanium alloy scaffolds (Figure 7A). *In vivo* staining of CD31, a marker of angiogenesis (Zhou et al., 2020), also showed that the best angiogenesis at 6 and 12 weeks after surgery occurred in the cTi/VEGF group (Figure 7B). Hence, the cTi/VEGF group augmented vascular regeneration of the local bone defect *in vivo*, and enhanced bone integration between the scaffold and the host.

## Discussion

Treating large bone defects is a major challenge in the medical field (Niikura et al., 2014). Autogenous bone grafting is common in clinic, but it can produce undesirable side effects like secondary operation, pain, and poor healing (Kuremsky et al., 2010). Bone tissue engineering combines materials science and biology to develop new ideal scaffolds that can replace autologous bone transplantation and promote bone integration. It contains biocompatible scaffolds and biological agents to simultaneously induce bone regeneration and simulate extracellular matrix.

Human cancellous bone was shown to have a density of 75–90% and a diameter of about 50–300 μm (Wang et al., 2017). Hence, we designed a scaffold with a porosity of 70% and diameter of 500 μm. Notably, excessively high porosity is not conducive to the maintenance of increased strength of the scaffold. We also chose a long diameter because a larger diameter can provide additional space for hydrogel attachment and material exchange, thus accelerating bone growth. The actual pore diameter of the composite scaffold can be reduced after the hydrogel is attached to the titanium surface, which makes the scaffold more physiological (Liu et al., 2016). According to previous studiy (Seliktar 2012), suitable pore size and porosity of hydrogel can promote nutrient transport, cell migration and proliferation. The thermosensitive collagen hydrogel used in this study has a pore size of 170 μm and a porosity of 40%, which is suitable for cell migration, adhesion and proliferation (Ansari et al., 2019). The porous scaffolds, arranged at random, not only provide a rough surface for cells to colonize, but also offer channels for nutrients to enter and metabolic waste to exit.

Drug release was shown to occur in an initial burst, before tapering off. The initial burst of drug release may be due to peripheral drug diffusion and leakage through pores. Therefore, pore size is a crucial factor in VEGF release. In fact, a large pore size was shown to have accelerated drug loss (Gao et al., 2011; Figueroa-Pizano et al., 2018). In the subsequent release phase, collagen degradation is likely responsible for VEGF release. The slow degradation of the gel promotes a slow release of VEGF at a more controlled rate. Based on our data, cTi/VEGF continuously releases VEGF over a 15-day period, and, is therefore, an excellent local delivery system for promoting angiogenesis and osseointegration. Swelling ability, reflecting water absorption capacity of hydrogel, is an important characteristic of tissue engineering hydrogel. The water absorption ability of hydrogel is mainly related to the content of hydrophilic groups. Good hydrophilicity can improve cell adhesion and promote bone tissue engineering repair (Li et al., 2016). The degradation of hydrogel lasted until 14 days. After 14 days, the remaining weight of the hydrogel almost unchanged, indicating that the degradation tends to be complete. The degradation rate did not reach 100%, probably due to the presence of hydrogel residues in the pores and difficult removal. The degradation rate of thermosensitive collagen hydrogel showed a homogeneous and slow characteristic, which can better achieve the sustained release of VEGF. In addition, the degradation of hydrogel in the scaffold pores also provides room for the bone ingrowth of porous interfaces of titanium alloys (Bai et al., 2020).

HUVECs proliferation in eTi was the slowest because of the lack of collagen and VEGF. The slow growth rate from the fourth till the seventh day of culture may be due to the limited pore plate area and cell contact inhibition. Based on our results, cTi/VEGF provided an excellent living environment for cells and promoted cell proliferation. VEGF serves as a survival factor for endothelial cells both *in vitro* and *in vivo* (Alon et al., 1995; Yuan et al., 1996). *In vitro*, VEGF prevents serum starvation-activated endothelial cell apoptosis, a process mediated by phosphatidylinositol 3-kinase (PI3K)/Akt pathway (Gerber et al., 1998; Fujio and Walsh 1999).

It was previously reported that VEGF mounted in collagen hydrogel can induce endothelial cells to recruit and form capillary-like structures, thus inducing three-dimensional vascular formation *in vitro* (Pepper et al., 1992). In addition, VEGF induces germination of rat aortic rings embedded in collagen gel (Nicosia et al., 1994). In some cases, VEGF delivery also induces lymphangiogenesis in mice (Nagy et al., 2002). In this study, the correlation between cTi/VEGF and angiogenesis *in vitro* was consistent with prior studies.

Generally, the endothelial cells migration ability determines the rate of early microvessel formation at the site of trauma. Faster migration speeds accelerate revascularization at the fracture site. In this study, we demonstrated that cTi/VEGF significantly accelerates HUVECs migration to the exposed area to completely cover the exposed surface and reduce the exposed area (Gao et al., 2011).

VEGF induces Bcl-2 (anti-apoptotic protein) expression and decreases Bax (pro-apoptotic protein) expression in HUVECs. *In vivo*, VEGF is intricately linked to animal development and the construction of vascular system. Hence, VEGF inhibition leads to extensive apoptotic changes in the vascular system of neonatal mice (Gerber et al., 1999). In addition, newly formed but not yet fully developed blood vessels in tumor tissues also exhibit severe VEGF dependence (Yuan et al., 1996; Benjamin et al., 1999). MMP-2 is a well-characterized angiogenic marker that is involved in the breakdown of type Ⅳ collagen, and is associated with endothelial cell migration and peri-cellular fibrinolysis (Presta et al., 2005). MMP-2 up-regulation suggests that cTi/VEGF significantly promote HUVECs maturation.

In this study, a cylindrical bone defect model was established in rabbit femoral lateral epicondyles, and various types of scaffolds were implanted into the defect site. Next, samples were retrieved at 6 and 12 weeks after implantation. Based on the Micro-CT results, the bone regeneration of cTi/VEGF was better than that of eTi and cTi, and the bone formation of eTi was of the smallest degree. The difficulty of introducing stent in the cTi/VEGF group was much greater than the other two groups, so the bone integration ability of the cTi/VEGF group was the strongest. At 6 and 12 weeks, the angiogenesis of the cTi/VEGF group was also significantly stronger than that of the other two groups. cTi/VEGF enhanced the ability of bone growth in the scaffold hole and bone integration around the scaffold. cTi/VEGF also made a valuable contribution to the stability of bone reconstruction.

In this study, we wrapped VEGF in collagen to prepare the surface coating of titanium alloy scaffolds. In addition, VEGF increased the bioactivity of the titanium alloy scaffolds without affecting the mechanical properties of the scaffolds. Our results confirmed that collagen hydrogels fitted with VEGF are a promising strategy for coating 3D titanium scaffolds. In future studies, we plan to increase the early stability of the thermosensitive collagen by adding bioactive substances, such as, cross-linking agents or sodium alginate. We also plan to prolong the release time of the drug.

## Conclusion

In this study, we enhanced osseointegration by introducing a surface bioactive coating to the scaffold. This accelerated HUVECs aggregation, blood vessels formation, as well as growth and osseointegration of the porous bone in the 3D printed titanium alloy porous scaffolds. We also evenly coated the thermosensitive collagen hydrogel with VEGF to fill the pores of the titanium alloy scaffolds and form a composite scaffold with good bioactivity and mechanical properties. The slow local VEGF release recruited endothelial cells, and enhanced vascular and bone regeneration and integration at the interface. Our results confirmed that the 3D printed porous titanium scaffold with VEGF/thermosensitive collagen hydrogel coating can provide an effective strategy for promoting angiogenesis and osseointegration.

## Data Availability

The raw data supporting the conclusions of this article will be made available by the authors, without undue reservation.
