# Supplementary material for: Sustained Release of VEGF to Promote Angiogenesis and Osteointegration of Three-Dimensional Printed Biomimetic Titanium Alloy Implants
Source: Front Bioeng Biotechnol. 2021 Nov 15;9:757767. doi: 10.3389/fbioe.2021.757767 (PMC8634467; doi:10.3389/fbioe.2021.757767)
Supplement: Supplementary file 1 [file DataSheet1.PDF]

Sustained Release of VEGF to Promote Angiogenesis and Osteointegration of Three-dimensional Printed Biomimetic Titanium Alloy Implantation

Youbin Li[1,2], Yuzhe Liu[1,2], Haotian Bai[1], Ronghang Li[1,2], Jing Shang[1], Zhengqing Zhu[1], Liwei Zhu[1,2], Chenyi Zhu[1], Zhenjia Che[1], Jincheng Wang[1,2], He Liu[1,2], Lanfeng Huang[1,*]

[1] Orthopaedic Medical Center, The Second Hospital of Jilin University, Changchun 130041, P. R. China

[2] Orthopaedic Research Institute of Jilin Province, Changchun 130041, P. R. China

* Corresponding author.

Email: jinchengwang@hotmail.com (JC Wang), heliu@jlu.edu.cn (H Liu), hlf@jlu.edu.cn (LF Huang)

Tel: +86 0431 8113 6572

Fax: +86 0431 8113 65

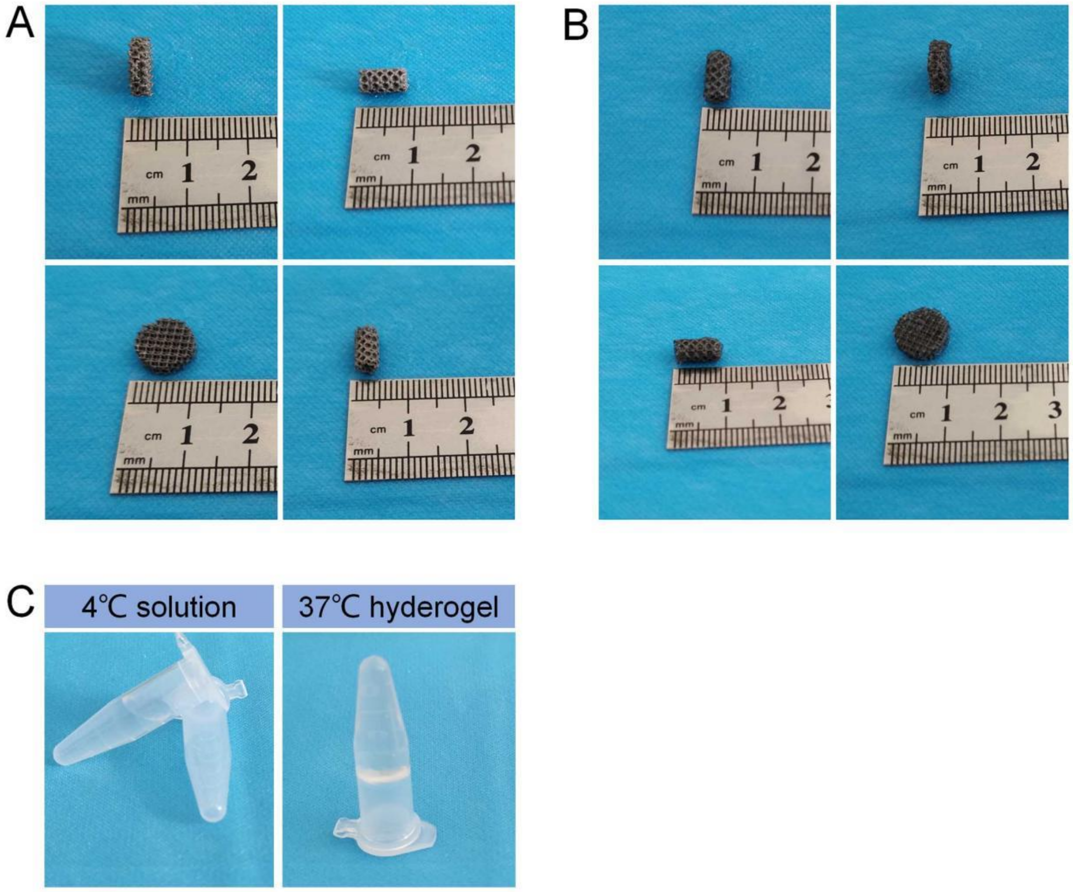

Fig. S1. (A) Characterization of disc-shaped and cylindrical eTi. (B) Characterization of disc-shaped and cylindrical cTi. (C) Transition of thermosensitive collagen hydrogels from liquid to gel state.

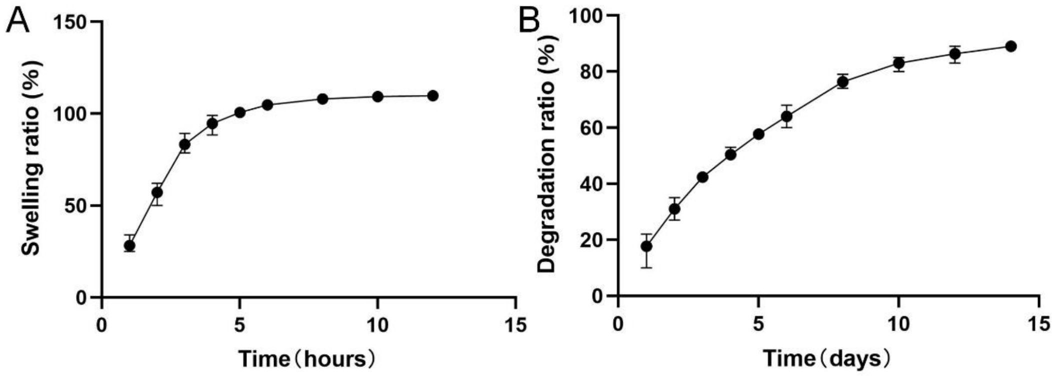

Fig. 3. (A) The swelling ratio curves of hydrogel measured in PBS at 37 °C. (B) The degradation curves of hydrogels measured in PBS containing 20 μg/mL collagenase I at 37 °C.

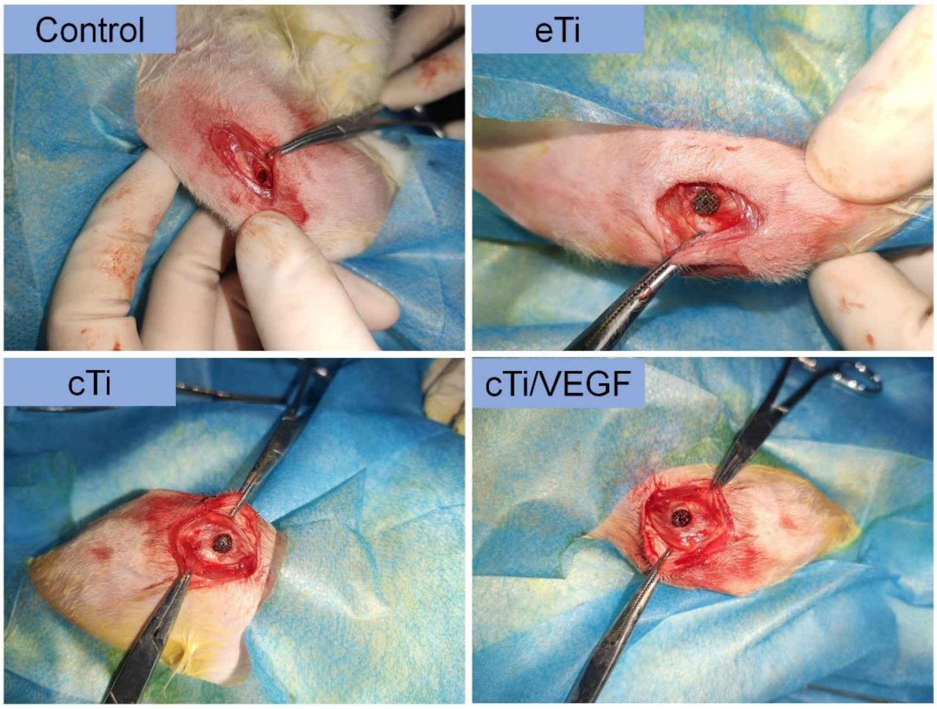

Fig. S3. The surgical procedure of scaffolds implantation.

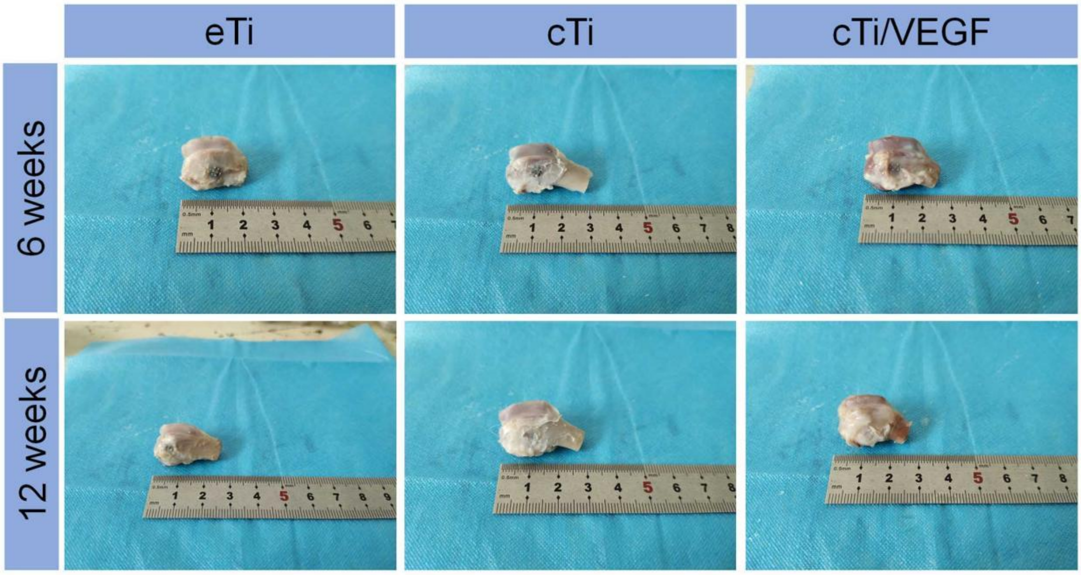

Fig. S4. Appearance of specimens obtained at 6 and 12 weeks.

Table S1 Primers used in RT-qPCR.

Gene	Forward primer	Reverse primer
GAPDH	GAGCACCAGAGGAGGACGA	TGGGATGGAAACTGTGAAGAG
MMP-2	GTGGA TGA TGCCTTTGCTCG	CCA TCGGCGTTCCCA TACTT
Bax	AGTAACATGGAGCTGCAGAG	AGTAGAAAAGGGCGACAACC
Bcl-2	ACTGGAGAGTGCTGAAGATTG	AGTCTACTTCCTCTGTGATGTTG
